# CX43 down-regulation promotes cell aggressiveness and 5-fluorouracil-resistance by attenuating cell stiffness in colorectal carcinoma

**DOI:** 10.1080/15384047.2023.2221879

**Published:** 2023-06-21

**Authors:** Yue Han, Haowei Wang, Hui Chen, Tianyuan Tan, Yiqing Wang, Hongjun Yang, Yanqing Ding, Shuang Wang

**Affiliations:** a Department of Pathology, Nanfang Hospital, Southern Medical University, Guangzhou, China; b Department of Pathology, School of Basic Medical Sciences, Southern Medical University, Guangzhou, China

**Keywords:** Connexin 43, colorectal cancer, chemoresistance, stiffness

## Abstract

Chemotherapy is one of the most commonly treatments of advanced colorectal cancer (CRC). However, the drug resistant following chemotherapeutic treatment is a significant challenge in the clinical management of CRC. Therefore, understanding the resistance mechanisms and developing new strategies for enhancing the sensitivity are urgently needed to improve CRC outcome. Connexins contribute to the formation of gap junctions among neighboring cells and then advance gap junctional intercellular communication (GJIC) for transportation of ions and small molecules. Although the drug resistance resulted from GJIC dysfunctional by aberrant expression of connexins is relatively well understood, the underlying mechanisms of mechanical stiffness mediated by connexin responsible for chemoresistance are largely unknown in CRC. Here, we demonstrated that connexin 43 (CX43) expression was downregulated in CRC and that loss of CX43 expression was positively correlated with metastasis and poor prognosis of CRC patients. The CX43 overexpressing suppressed CRC progression and increased the sensitivity to 5-fluorouracil (5-FU) *via* enhanced GJIC *in vitro* and *in vivo*. Moreover, we also highlight that the downregulation of CX43 in CRC increases the stemness of cells via reducing the cell stiffness, thus promoting the drug resistance. Our results further suggest that both effects, that is changes in the mechanical stiffness of the cell and GJIC mediated by CX43 deregulated, are closely related to drug resistance in CRC, which indicating CX43 as a target against cancer growth and chemoresistance in CRC.

## Introduction

Colorectal cancer (CRC) is one of the commonly diagnosed cancers worldwide^1^. The initial events in CRC tumorigenesis have been relatively well-studied and therapeutic advances over the past decades; however, the metastatic CRC (mCRC) patients account for about 50% of newly diagnosed cases, which represent a critical challenge for CRC therapy^2^. Chemotherapy is a commonly used therapeutic means for mCRC. 5-Fluorouracil (5-FU) is the main of first-line chemotherapeutic strategy and has high tumoricidal activity against advanced CRC. However, the effectiveness is often reduced by the development of drug-resistance after prolonged 5-FU treatment^3^. Therefore, understanding the molecular mechanisms of chemotherapy resistance and developing new strategies for enhancing the sensitivity and preventing drug-resistance are urgently needed to improve CRC outcome.

The effects of the extracellular matrix (ECM) stiffness in chemotherapy resistance were predominantly investigated^4^. Growing studies demonstrated that the resistance to drugs, including cisplatin, paclitaxel and 5-FU, depended on ECM stiffness^4,5^. However, emerging data also showed the role of cell stiffness in resistance in tumor^6–9^. The softer tumor cells showed an alteration to drug resistance by multiple mechanisms in different tumor entities, including hepatocellular carcinoma^6^, leukemia^7^, ovarian cancer^8^, and prostate cancer^9^. However, much less is known about the role of cell mechanical stiffness in CRC chemotherapy resistance. Therefore, investigating the mechanical properties of CRC cells may to better understand the mechanisms responsible for cancer drug-resistance.

Gap junctions composed of connexin proteins are membrane channels found in all human cells^10^ and directly transfer small molecules or electrical charge among neighboring cells, which are responsible for various physiological and pathological events including cell growth, differentiation, and damage^11^. There are 21 connexin isoforms in humans. Among these connexin proteins, connexin 43 (CX43), with molecular weight of 43 kDa, is the most abundant, and its expression is aberrant in various cancers depending on the types and stage of cancer^12,13^. The deregulation of CX43 expression level has been shown to associate with cancer recurrence, spread, and resistance to drugs. For instance, the overexpression CX43 in glioblastoma cells displayed chemoresistance to temozolomide, a DNA-alkylating chemotherapeutic agent^14^. More recently, work by Arora, *et al*. has demonstrated the positive effect of CX43 through formatting gap junction intercellular communication (GJIC) on increasing cisplatin cytotoxicity by inducing DNA damage in lung and ovarian cancer cell lines^15^. In CRC, CX43 knock-down could affect chemotherapeutic agent-induced cytotoxicity in a cell density-dependent manner^16^. These results supported the importance of gap junction in cancer biology and drug resistance^17^. However, up to now, the precise mechanisms by which CX43 contributed to chemoresistance are largely unknown, especially CRC. Here, we investigated the role of CX43 on the cell growth and chemosensitivity in CRC and uncovered the underlying mechanisms, which might benefit the development of future therapies for CRC.

## Materials and methods

### Tissue specimens and cell lines

For this study, the tissue specimens were obtained with informed consent from the ethics committee of Nanfang Hospital, Southern Medical University (Guangzhou, China). All CRC tissue samples were obtained from primary CRC patients who underwent elective surgery in Nanfang Hospital, Southern Medical University. Fresh CRC and paired non-tumor mucosal tissues were used for real-time PCR and western blotting. Moreover, formalin-fixed tumor tissue specimens, comprising 101 CRC tissues and 90 adjacent non-tumor tissues, were collected and used to investigate the expression of CX43 by immunohistochemistry (IHC). No patient received any chemotherapy and radiotherapy before operation.

The immortalized colon mucosa epithelial cell line (FHC) and human CRC cell lines, including SW480, SW620, LoVo, LS174t, RKO, DLD1, HCT116, and HT29, were obtained from the ATCC. A subclone named M5 with enhanced metastatic abilities in liver was isolated by *in vivo* selection of SW480 cells in our previous studies^18^. All CRC cell lines were cultured in RPMI 1640 medium (Invitrogen, Carlsbad, CA, USA) with 10% fetal bovine serum (FBS, Invitrogen). FHC cells were cultured in DMEM:F12 medium (Invitrogen) with 10% FBS. All cell lines were cultured at 37°C and in 5% CO_2_ in air in a humidified incubator.

### RNA isolation and quantitative real-time RT-PCR

RNA extraction from tissues and cell lines with TRIzol Reagent (TaKaRa, Dalian, China) followed the manufacturer’s protocol. cDNA was generated with PrimeScript RT Reagent Kit (TaKaRa). Real-time PCR was carried out using TB Green Premix Ex *Taq*™ (TaKaRa). The reaction conditions of PCR were as follows: 95°C for 30 s, followed by 40 cycles of amplification (95°C for 5 s, 55°C for 30 s, and 72°C for 40 s), and then 72°C for 10 min. β-actin was used as an internal quantitative reference. The assays were performed in triplicate for each case to allow the assessment of technical variability. Comparative quantification was determined using the 2^−ΔΔCt^ method. The primer sequences are listed in Supporting Information Table S1.

### Western blotting analysis

Cells and tissues were lysed by RIPA buffer containing protease and phosphatase inhibitors and PMSF. The proteins were separated by 10% SDS-PAGE and transferred onto polyvinylidene fluoride (PVDF) membranes (Millipore, Darmstadt, Germany). After blocking for 1.5 h in 5% skimmed milk diluted by 1 × PBS-T (0.5% Tween−20), the membranes were incubated overnight at 4°C with the following primary antibodies: anti-CX43 (ab11370, Abcam, MA, USA), anti-CD133 (abs131197, Absin, Shanghai, China), anti-CD44 (DF6392, Affinity Biosciences, OH, USA), anti-Nanog (AF5388, Affinity Biosciences), anti-SOX2 (ab92494, Abcam), anti-cleaved caspase-3 (AF7022, Affinity Biosciences), anti-cleaved caspase-9 (AF5240, Affinity Biosciences), anti-caspase-3 (AF6311, Affinity Biosciences) or anti-caspase-9 (AF6348, Affinity Biosciences). Anti-GAPDH (Proteintech Group, Wuhan, China) was used as protein-loading controls. Blots were incubated with HRP-conjugated secondary antibodies for 1 h at room temperature and visualized with ECL Western Blotting Substrate (ThermoFisher Scientific, IL, USA).

### IHC analysis

IHC was carried out as previously described^18^. The slides were incubated overnight with anti-CX43 antibody (1:500, Abcam) at 4°C. Immunodetection was performed using diaminobenzidine (DAB) (Aligent, CA, USA). The reaction time of each section was consistent, followed by counterstaining with hematoxylin.

IHC-stained scores were determined based on both the intensity and proportion of CX43-positive cells, as previously described^18^. According to the percentage of the positive stained areas in relation to the entire section, extent of staining was scored on a scale of 0–4 as follows: 0% (0); 1%−25% (1); 26%−50% (2); 51%−75% (3); and 76%−100% (4). Staining intensity was scored on a scale of 0–3 as follows: negative (0), weak (1), medium (2), or strong (3). The summation of the staining-extent and staining-intensity scores was regarded as the final score for CX43 (on a scale of 0–7). A final staining score of ≥3 was considered to indicate high level of CX43.

### Construction of cell lines with stably overexpressing CX43

The full-length human CX43 with 1149-bp DNA fragment amplified by PCR and cloned into GV260 lentiviral vector (Genechem, Shanghai, China). Virus particles were harvested 48 h after GV260-CX43 transfection cells using the recombinant lentivirus-transducing units. The empty lentiviral vector GV260 was used as the control.

### Construction of cell lines with downregulated CX43

The siRNA sequences with specifically targeting CX43 and a negative control siRNA were designed and synthesized (GenePharma, Shanghai, China, Supporting Information Table S2). The most effective siRNA sequence (1^#^ and 3^#^) in achieving knockdown of CX43 was demonstrated by real-time PCR and Western blotting. HCT116 cells were infected with the lipofectamine 2000 reagent (ThermoFisher Scientific).

### *Function assays* in vitro

Cell proliferation, colony formation, wound-healing, invasion assays, flow cytometry cell cycle, and cell apoptosis were performed *in vitro* according to standard protocols, as described previously^19^. Details are described in Supporting Information. All experiments were performed in triplicate.

### Dye-transfer assay

Fluorescent dye-transfer assays which determine the levels of adhesion and communication among cells were used to estimate the function of gap junctions by flow cytometry. Calcein-AM (Dojindo, Kumamoto, Japan), a membrane permeable fluorescent dye, is trapped within the cell and enabled pass to adjacent cells by functional gap junctions. A total of 1.5 × 10^5^ CRC cells were seeded in 6-well plates and incubated for 24 h before co-culture. On the next day, after washing with PBS, cells to be labeled (donor cells) were incubated in RPMI medium for 2 h with 2 μM Calcein-AM. Next, cells were washed twice with PBS to remove free dye and non-de-esterified dye, and then were incubated in serum-free RPMI medium for 30 min at 37°C. The dye donors were then co-cultured with dye acceptors (unlabeled cells) at a ratio of 1:1 at 37°C for the indicated incubation times. Co-cultures were trypsinized, fixed in 4% formaldehyde and analyzed by flow cytometry. Labeled and unlabeled cells without any treatment were used as control. The increase in MFI of unlabeled cells indicated increased dye transfer through gap junctions.

### Determination of drug sensitizing by CCK-8 assay

A total of 1 × 10^3^ cells were seeded in 96-well plates and cultured for 24 h, which would be subjected to a range of 5-FU (Sigma-Aldrich) concentration (1, 4, 8, 16, 32, and 64 μg/mL). Where indicated, 20 μM fresh-dissolved Oleamide (OL, Sigma-Aldrich), an inhibitor for gap junction, was added in culture media 24 h before 5-FU stimuli. 5-FU was freshly dissolved in PBS at stock solutions. After the cells were exposed to Oleamide and 5-FU for 48 h or 72 h, it would be treated with fresh medium with CCK-8 solution for 4 h. The plates were read on a microplate reader using a wavelength of 450 nm.

### Immunofluorescence microscopy

The CRC DLD1 cells were evenly seeded into the confocal dishes. When the cells in the dishes grew to an appropriate density followed by washing with PBS. Then, the cells were fixed using 4% paraformaldehyde at 4°C for the overnight storage, and after permeabilization with 0.1% TritonX−100/PBS, 5% BSA/PBS was used to block for 1 h. Then, the dishes were incubated with mouse anti-α-tubulin antibody (1:100, Proteintech Group) overnight at 4°C. Samples were stored at room temperature for 0.5 h, next washed three times in PBS and incubated Alexa Fluor 594 conjugated secondary antibody (1:100, ZF−0513, ZSGB-BIO, Beijing, China), and then kept in dark place at room temperature for 1 h. Nuclear stained with DAPI for 5 min (C0065, Solarbio, Shenzhen, China). Finally, the samples were imaged by Olympus confocal microscope. In addition, permeabilization and blocking were omitted when staining with Phalloidin (1:100, Cytoskeleton, Shanghai, China). It can be directly dyed in dark at room temperature for 1 h, and then re-stained with DAPI to take photos.

### Animals

Balb/C-nu/nu nude mice (3–4 weeks old) were obtained from the Laboratory Animal Center at the Southern Medical University. The protocol was approved by the Committee on the Ethics of Animal Experiments of Southern Medical University. The animals were fed with an autoclaved laboratory rodent diet. The animals were randomized into four groups: control, 5-FU, CX43 and CX43 + 5-FU. For tumorigenicity assay, a total of 1 × 10^7^ CRC with CX43-overexpression and control cells were subcutaneously injected into the flank of the nude mice (*n* = 10). When the macroscopic tumor was visible, two groups of nude mice were randomly set as control or treatment groups. Control group was injected intraperitoneally with PBS (untreated control and CX43) and the treatment groups were intraperitoneally injected with 5-FU (25 mg/kg, 5-FU and CX43 + 5-FU). The drug or PBS was administered by intraperitoneal injection every other day, and the length, width, and height of tumors were measured and recorded meanwhile. After 3 weeks of monitoring, the mice were sacrificed by cervical dislocation. The tumors were separated and weighed and fixed in 10% neutral-buffered formalin. Tumor volume was calculated according to the formula: V (volume) = (D × d^2^)/2 (D indicates the longest diameter and d indicates the shortest diameter).

### Tumor sphere formation assays

The mixture culture medium was compounded and included 20 ng/mL basal fibroblast growth factor (PeproTech, Rocky Hill, NJ, USA), serum-free 1640 medium (Invitrogen), 20 ng/mL epidermal growth factor (PeproTech), 4% BSA (Sigma), 2% B−27 Supplement (Invitrogen), and 5 μg/mL insulin (Sigma-Aldrich). DLD1 was digested and resuspended with the mixed medium. Each hole in the 6-hole ultra-low attachment plate was seeded with 1 × 10^3^ cells and placed in the incubator at 37°C in a 5% CO_2_ saturation humidity for about 20 d. The number and size of spheres were observed and counted under the microscope. When cell mass >2 × 10^3^ cells were defined as tumor sphere.

### Atomic force microscopy (AFM)

Slides were fixed with 4% paraformaldehyde under ultraviolet lamp for 1 h in advance, washed with PBS and dried in the air. The cells in logarithmic growth phase were digested routinely, one drop was put on one slide, and the other slide was used to push the slide until the cells were monolayer. The colloidal probe nanoindentation experiments were performed with AFM (Bioscope Catalyst, Bruker). The measurements were accomplished using spherical tips made of non-conductive silicon nitride (5 μm radius). The nominal spring constant of the cantilever (MLCT-D, Bruker) was 0.06 N/m. Hertz model was used to estimate the contact point and the apparent Young’s modulus on the force curve.

### Statistical analysis

Statistical analysis was conducted by GraphPad Prism 7.0 (GraphPad Software Inc.) package. The experimental data were summarized and analyzed using *t*-test and Chi-square test. Survival curves of CX43 expression of CRC patients were obtained using the Kaplan–Meier and evaluated by log-rank testing. *P* < .05 was defined as statistically significant.

## Results

### CX43 is downregulated in CRC and predicts a poor prognosis in patients with CRC

**Figure 1.**
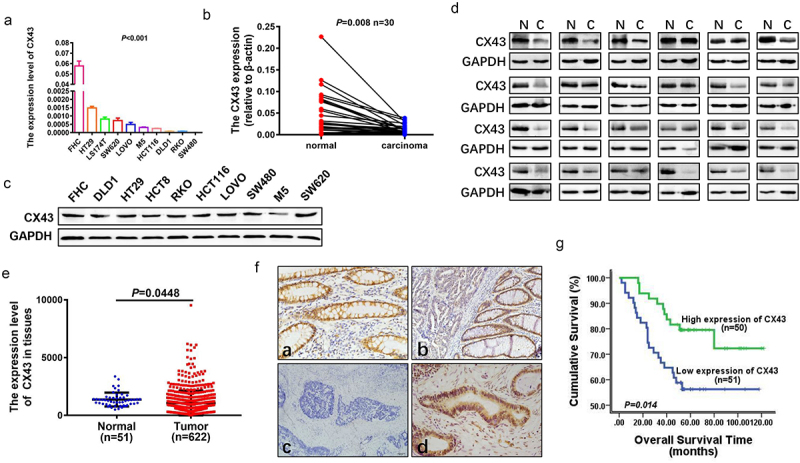
CX43 is down-regulated in CRC and may be a predictor for poor prognosis of CRC patients. (a and b) Expression of CX43 in human CRC cells and colon mucosa epithelial (FHC) cells (a) and in CRC tissues and the matched adjacent normal tissues (b) by real-time PCR analyses. (c and d) Expression levels of CX43 protein in CRC cell lines (c) and human CRC tissue samples (d) by western blot. (e) Analyses of CX43 expression in 622 CRC patients and 51 normal colorectal population in TCGA database. (f) Representative images of CX43 expression in CRC and adjacent normal tissues by IHC. (a) Positive expression of CX43 in normal colorectal mucosa. (b) Weak expression of CX43 in a tumor tissue sample and high expression of CX43 in its normal mucosal counterpart were observed in one field of a tissue sample from a single patient. (c) Negative expression of CX43 in CRC tissue. (d) High expression of CX43 in CRC tissue. (g) Correlation between CX43 expression and overall survival in CRC patients. The median value of CX43 expression was chosen as the point for separating tumors into low-level and high-level expression groups (log-rank *P* = .014).

To estimate the expression level of CX43 in CRC, the expression levels of CX43 mRNA was firstly determined in CRC cell lines and tissue samples. As shown in Figure 1a, there was high expression of CX43 in FHC cells, an immortalized cell line. However, all CRC cell lines showed a low-level expression of CX43 mRNA. Furthermore, relatively low level of CX43 was founded in CRC tissues compared with non-tumor tissues of the same donor (Figure 1b). Western blot analysis also showed that the levels of CX43 protein were decreased in most CRC cell lines and CRC tissues, consistent with the results from RNA levels (Figure 1c,d). These are consistent with the data available from TCGA database (Figure 1e).

Based on the above results, we then detected CX43 protein levels by IHC in 101 CRC and 90 adjacent normal mucosa tissues. CX43 was highly expressed in 76.67% (69/90) of normal mucosa tissues; however, 63.3% (64/101) of CRC tissue samples had relatively low level of CX43 expression (Figure 1f). The expression of CX43 was significantly downregulated in CRC tissues (*P* = .046). The relationships between CX43 protein expression and clinicopathological characteristics are summarized in Table 1. Low CX43 expression was positively correlated with metastasis (*P* = .023) but not correlated with other clinicopathological features. We next want to ask whether CX43 expression is associated with clinical progression of the patients with CRC. The results from the clinical samples demonstrated that CRC patients with lower CX43 levels had shorter overall survival times (log-rank *P* = .014, Figure 1g). In general, these data showed that CX43 expression is downregulated in CRC, and the low CX43 is a poor prognostic biomarker for patients with CRC.

**Table 1.** Correlation between the clinicopathological features and expression of CX43.

Characteristics	n	CX43 expression		
		Low (%)	High (%)	*P* value
**Gender**				
Male	63	33 (52.4)	30 (47.6)	0.625
Female	38	18 (47.4)	20 (52.6)	
**Age (years)**				
<57	25	13 (52.0)	12 (48.0)	0.862
≥57	76	38 (50.0)	38 (50.0)	
**Tumor site**				
Proximal colon	34	15 (44.1)	19 (55.9)	0.434
Distal colon	16	7 (43.8)	9 (56.2)	
Rectum	51	29 (56.9)	22 (43.1)	
**Tumor size (cm in diameter)**				
<5	59	28 (47.5)	31 (52.5)	0.469
≥5	42	23 (54.8)	19 (45.2)	
**Tumor differentiation**				
Good	25	9 (36.0)	16 (64.0)	0.053
Moderate	50	24 (48.0)	26 (52.0)	
Poor	26	18 (69.2)	8 (30.8)	
**T-stage**				
1–2	15	10 (66.7)	5 (33.3)	0.224
3	74	37 (50.0)	37 (50.0)	
4	12	4 (33.3)	8 (66.7)	
**N-stage**				
0	64	32 (50.0)	32 (50.0)	0.530
1–2	37	19 (51.4)	18 (48.6)	
**M-stage**				
0	96	46 (47.5)	50 (52.5)	0.023
1	5	5 (100.0)	0 (0.0)	

### *CX43 represses CRC cell growth and induces apoptosis* in vitro

**Figure 2.**
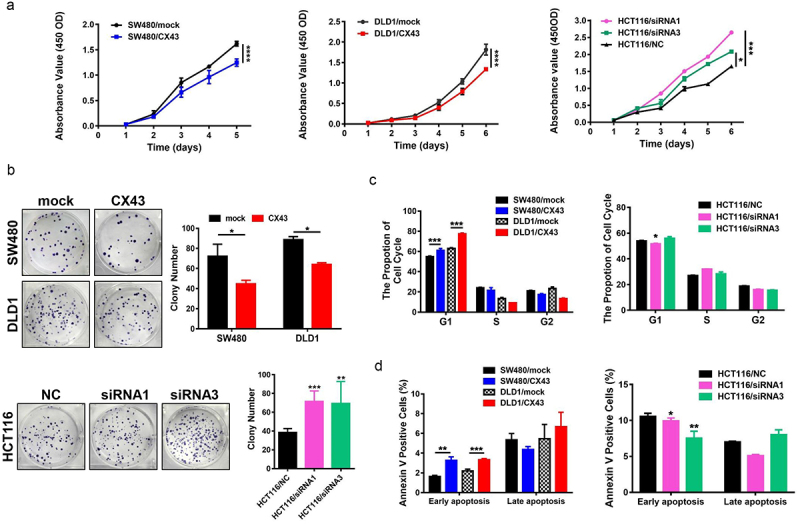
CX43 restrains the proliferation and induces cell cycle arrest and the apoptosis of CRC cells *in vitro*. (a) CX43 overexpression repressed cell proliferation of SW480 and DLD1 cells, while, Knock-down of CX43 promoted cell proliferation of HCT116 by CCK − 8 assays. (b) CX43 decreased the colony formation capacity of CRC cells. Experimental images (left) and statistical analysis (right) are shown. (c) CX43 overexpression induced G1 phase cell cycle arrest in CRC cells. (d) CX43 promoted early apoptosis in CRC cells. Data are presented as the mean ± SD. The results were reproducible in three independent experiments. *P < .05, **P < .01, ***P < .001, and ****P < .0001.

To determine whether CX43 downregulation plays a causal role in CRC carcinogenesis and progression, two CRC cell lines with CX43-overexpressing were established. Moreover, we also knocked down the endogenous expression of CX43 by siRNA transfection in HCT116 cells. Real-time PCR and western blot analysis confirmed that CX43 expression was successfully up-regulated or down-regulated in the corresponding CRC cells (Supporting Information Figure S1). A significantly slower proliferation was observed in CX43-overexpressing SW480 and DLD1 cells compared with mock cells (Figure 2a). The inhibit effect of CX43 overexpression on cell proliferation was also determined by the colony formation (Figure 2b). In contrast, the downregulation of CX43 promoted proliferation and colony formation in CRC cells (Figure 2a,b).

We next studied the molecular mechanisms by which CX43 affects proliferation. Flow cytometry assays confirmed that CX43 overexpression could increase the percentage of G1-phase cells in both CX43-overexpressing CRC cells (SW480 cells, *P* = .002 and DLD1 cells, *P* = .001, Figure 2c and Supporting information Figure S2). We also evaluated the effects of CX43 on cell apoptosis in CRC cells. The upregulation of CX43 expression increased the percentage of early apoptosis (SW480, *P* = .0016 and DLD1, *P* = .0007, Figure 2d and Supporting information Figure S2) in both CX43-overexpressing cell lines compared with controls. Conversely, CX43 depletion mediated by two siRNA in HCT116 cells induced the opposite results (Figure 2c,d). In short, these data indicate that the CX43-mediated inhibition in cell proliferation is modulated by G1-S checkpoint and apoptosis.

### *CX43 inhibits CRC cell migration and invasion* in vitro

**Figure 3.**
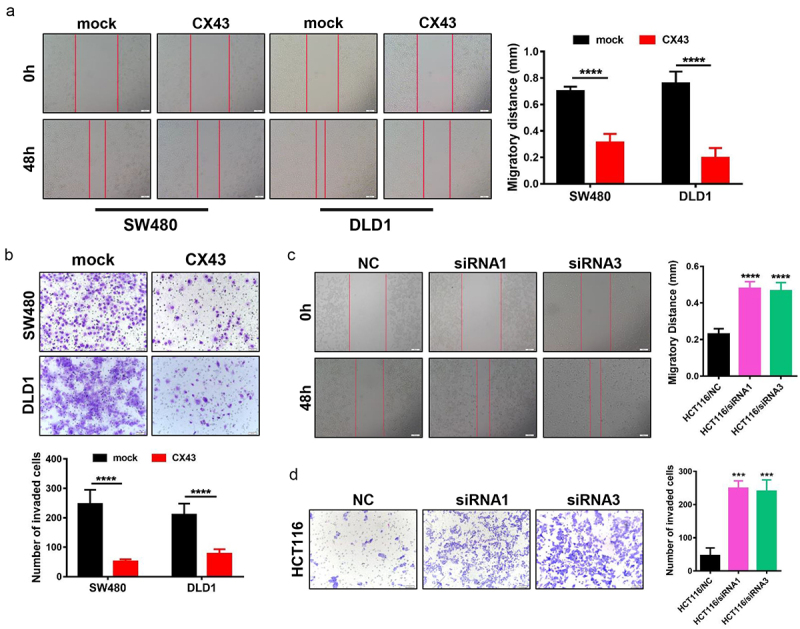
CX43 repressed the cell migration and invasion of CRC cells *in vitro*. (a) The migration ability was decreased in CRC cells with CX43 overexpressiong, as indicated by the wound healing assay. (b) Matrigel invasion chamber assays showed that CX43 overexpression inhibited the invasiveness. (c and d) CX43 depletion mediated by siRNA promoted cell migration (c) and invasion (d). Scale bars, 100 μm. Each experiment was conducted at least in triplicates, and the data are indicated as mean ± SD.

The role of CX43 in cell migration and invasion was examined by scratch-wound healing and Transwell assays. As shown in Figure 3a, after enforcing the expression of CX43, the migratory of SW480 and DLD1 cells were both remarkably impaired (*P* < .0001). Meanwhile, the CX43 elevation in both CRC cell lines significantly inhibited cancer cell invasion through Matrigel extracellular matrix (*P* < .0001, Figure 3b). However, the knockdown of CX43 expression significantly increased cell migration and invasion (Figure 3c,d). The results suggested that CX43 overexpression inhibited cell migration and invasion in CRC.

### *CX43 overexpressing restores 5-FU-sensitivity of CRC cells in vitro and* in vivo

Given, CX43 contributes to formation of gap junctions among neighboring cells and thus facilitates GJIC, we next studied the GJIC competence of CX43-overexpressing CRC cells by a dye-loading technique combined with flow cytometry. The Calcein-AM dye transfer is a commonly employed method to detect the presence of functional GJIC^20^. We performed Calcein-AM dye-transfer analysis and the results showed a significant increase in cellular communication both in two CX43-overexpressing CRC cells, which indicated CX43 overexpression increase GJIC (Supporting information Figure S3).

**Figure 4.**
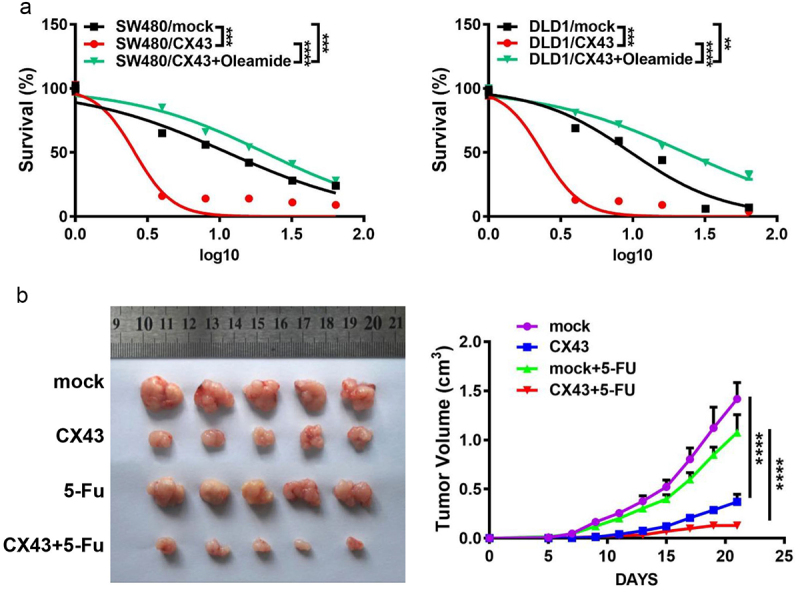
CX43 overexpression increased sensitivity of CRC cells to 5-FU. (a) CX43 overexpression improved the sensitivity of CRC cells SW480 and DLD1 to 5-FU. Oleamide, a gap junction inhibitor, restricted the elevated sensitivity of CRC cells with CX43 overexpression to 5-FU. (b) Subcutaneous tumorigenesis experiment by injection of DLD1/CX43 and DLD1/mock cells in nude mice. They were randomly divided into two groups and with/without 5-FU treatment respectively for about 21 d. Tumor volumes were measured regularly. Data points are the mean tumor volumes ± SD.

Increasing evidence has suggested that the anti-neoplastic agent’s cytotoxicity can be enhanced by modulating GJIC^21^. These reports prompted us to test if CX43 overexpression affects 5-FU sensitiveness in CRC cells. We observed that the both cytotoxicity of 5-FU and cisplatin increased with an increase of CX43 expression level (Figure 4a and Supporting information Figure S4). Herein, the IC50 to 5-FU was 11.310 μg/mL and 9.709 μg/mL in mock SW480 and DLD1 cells, respectively, however, the overexpressing CX43 decreased IC50 to 5-FU to 2.579 μg/mL in SW480 and to 2.355 μg/mL in DLD1 cells. Moreover, our data showed that adding Oleamide, a gap junction inhibitor, could abolish the sensitivity of CRC cells to 5-FU induction by CX43 overexpression, indicating the requirement of GJIC for chemosensitivity modulating by CX43 (Figure 4a and Supporting information Figure S4).

Furthermore, to investigate the antitumor effect of CX43 alone or in combination classical chemotherapy drug, DLD1 cells with overexpressing CX43 or control cells with/without 5-FU treatment were subcutaneously injected into each flank of nude mice to established xenograft tumors. The results showed that both CX43 ectopic overexpression and 5-FU treatment suppressed the tumor growth compared with mock cells (Figure 4b). Importantly, the combination of CX43 overexpressing and 5-FU treatment has a significant inhibitory effect on tumor growth in the subcutaneous tumor model, compared with CX43 overexpressing or 5-FU along (Figure 4b).

### CX43 enhances cell stiffness via polymerizing cytoskeleton in CRC cells

**Figure 5.**
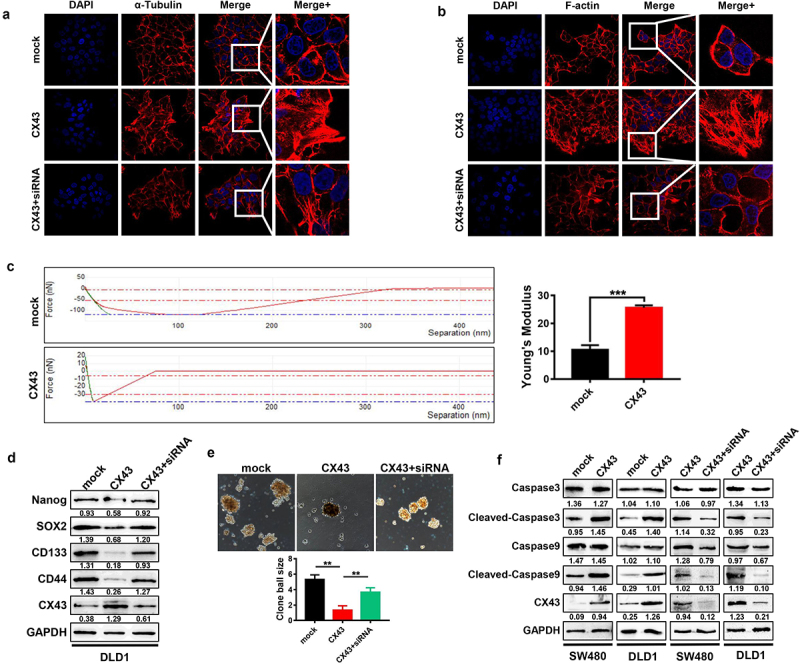
CX43 increased cell stiffness by polymerizing CRC cytoskeleton. (a and b) Confocal microscopy analysis of α-tubulin polymerization and F-actin cytoskeletal remodeling in CRC cells with CX43 overexpression and knock-down. (c) The young’s modulus of CX43 overexpressed DLD1 cells was measured by atomic mechanics microscope. Mechanical curve (left) and quantitative analysis (right) are displayed. (d) Western blot analyses expression of stem cell characteristic related proteins in CRC cells with CX43 overexpression. (e) The tumor cell spheroidizing ability of CX43 overexpression cells was detected by tumor sphere formation assays. (f) Western blot was used to survey the relative expression of apoptosis pathway related proteins in CX43 overexpression cells. The downregulation of CX43 could rescue the above phenotypes (d, e and f).

Other than GJIC-mediated bystander effects which diffuse antitumor compounds to neighboring cells, some studies reported that connexins mediated signaling via their C-terminal tails interacting with signaling mediators^22^. It has been shown that CX43 plays important roles in regulating cytoskeleton rearrangements by binding with microtubules (consisting of α/β-tubulin) and actin^23–27^. Using confocal microscopy, we observed that CX43 overexpressing could induce α-tubulin and F-actin polymerization: the stress fibers looked thicker, and bundles appeared more parallel to each other than in mock cells, whereas the down-regulated of CX43 mediated by siRNA disrupted α-tubulin and F-actin polymerization (Figure 5a,b).

Since the dynamic changes in the cytoskeleton are associated with cell stiffness, we wondered whether the organization of the cytoskeleton by CX43 would impact on the biomechanical properties of CRC cells. Thus, we used AFM to compare the stiffness of CX43-overexpressing DLD1 cells and mock cell populations. The Young’s modulus of DLD1 cells is shown in Figure 5c. The average Young’s modulus of DLD1 cells was 10.530 ± 0.9412 nN. The average Young’s modulus of DLD1 cells with CX43 overexpression increased 2.5-fold to 25.630 ± 0.491 nN (*P* < .001). Altogether, these results strongly suggest an unexpected functional link between CX43 expression and cell stiffness.

### *CX43 suppresses stemness and promotes apoptosis* in vitro *by enhancing cell stiffness*

The cancer stem cells (CSCs) have been found to be responsible for the drug resistance, recurrence, and metastasis^28^. In recent studies, cell softness has been found to regulate tumorigenicity and stemness of cancer cells^29^, which indicated that the mechanical softness represents an essential characteristic for CSCs. Next, we asked whether CX43 affects the stem-like characteristic of CRC cells. Thus, we examined the effects the C×43 on the stemness of CRC cells. When the cell stiffness is increased mediated by C×43 overexpression, the expression of stemness biomarkers was downregulated in CRC cells (CD133, CD44, Nanog, and SOX2, Figure 5d), and the sphere-forming capacity of CRC cells also was suppressed (Figure 5e), while the down-regulated of CX43 mediated by siRNA reversed this effect (Figure 5d,e). Stem cell-like cancer cells usually express high levels of anti-apoptotic proteins to help their survive the drug attack^30^. The stiffness of a cell is also associated with the apoptotic response of cells^7^. In the first part of this study, flow cytometry assays revealed that CX43 overexpression induced cell early apoptosis of CRC. In addition, the western blotting also showed that CX43 overexpression increased the activation of caspase-3 and caspase-9 in CRC cells (Figure 5f), suggesting that CX43 triggers apoptotic pathways along with attenuating cell stemness in CRC. Together, these data suggest that the downregulation of CX43 in CRC increases the stemness of cells by reducing the cell stiffness, thus promoting the drug resistance.

## Discussion

Connexins are a family of transmembrane proteins, and each can form homoetric channels or heteromeric hemichannels with the same or different connexin isoforms, allowing the direct exchange of small substances with a molecular weight of up to 1.5 kDa^31^. Thus, aberrant connexin expression can alter gap junction and contribute to cancer development and progression^32^. Although the expression and functional studies have identified connexins and their derived GJIC as potential tumor suppressor, it has been made more complex by recent growing evidence that connexins contributing to malignant progression in some cancer late stages by heterocellular GJIC between cancer cells with adjacent cells^33–35^, as well as the functions that are independent of their GJIC function. CX43 is one of the most studied connexin members in cancer and is frequently downregulated or expressed in the wrong location in tumors, resulting in loss of GJIC to play inhibitory roles in tumorigenesis^22,36,37^. However, increased expression of CX43 was reported in multiple cancers, including breast cancer^38^, gastric cancer^39^, hepatocellular carcinoma^40^, and CRC^41^. In a study of hepatocellular carcinoma, CX43-silenced HuH7 cells showed lower growth and higher differentiation, however, CX43-overexpressing cells exhibited rapid growth and low differentiation^40^. Han *et al*. reported a progressive increase in cytoplasmic CX43 expression from colon normal epithelium to tubular adenoma/severe dysplasia and to carcinoma, indicating its oncogenic role in colonic neoplastic progression^41^. Additionally, they found that CX43 was relatively increased at the invasive tumor front in all stages of CRC. Therefore, the complexities that are associated with CX43 in the processes of tumorigenesis, prognosis prediction, and progression in CRC remain unresolved. In the present study, we demonstrated that CX43 expression was downregulated in CRC cell lines and tissue samples, and that loss of CX43 expression was positively correlated with CRC metastasis and poor prognosis of CRC patients. These observations are consistent with previous study from Sirnes *et al*. who also found the decreases of CX43 expression during CRC development is associated with reduced patient survival^22^. In addition, we demonstrated that the ectopic overexpressing CX43 suppressed cell growth and induced apoptosis in CRC *in vitro* and *in vivo*. Meanwhile, our results confirmed that the downregulation of CX43 by small interfering RNA resulted in more aggressive growth of CRC cells. Together, the experimental evidence indicates that CX43 may act as tumor suppressor in CRC.

GJIC allows the exchange of second messengers and small molecules such as Ca^2+^ between cells. GJIC is formed by the docking of two hemichannels on the cellular membrane, and each hemichannels is composed of six connexins^42^. As a result, antitumor drugs and toxic metabolites were found to diffuse to neighboring cells via GJIC, inducing cancer cell death^43^. This phenomenon has been called the bystander effect^44^. Indeed, CX43 expression has been reported to increase cancer cell permeability to chemotherapeutics^16,45–49^. For example, greater drug permeability and higher chemosensitivity were observed with increasing of CX43 expression in breast cancer MCF−7 cells^45^. The overexpressing CX43 in glioblastoma cells could elevate significant permeability to paclitaxel and doxorubicin^46^. Similarly, in CRC, CX43 can sensitize CRC cells to paclitaxel^47^, oxaliplatin^49^, and 5-FU^16^ by enhancing the bystander effect. Indeed, we identified that CX43 overexpressing enhanced GJIC in CRC cells, thereby increasing CRC cell sensitivity to 5-FU and cisplatin, and that Oleamide, a gap junction inhibitor, could restore chemosensitivity by reducing CRC cell death. The results of the present study also supported this conclusion that CX43 overexpression can facilitate targeted drug delivery to cancer cells *via* enhanced drug permeability.

Other than GJIC-mediated bystander effects which diffuse antitumor compounds to neighboring cells, some studies reported GJIC-independent roles of connexins^50^. We also assume that the bystander effect mediated by GJIC is not only mechanism by which CX43 exerts its modulatory effects on drug-sensitivity. CX43, an old story, may have new functions and mechanisms for drug-resistance in cancer. Substrate stiffness of the extracellular matrix (ECM) has important functions in numerous physiological and pathological processes^51^. Changes in the ECM composition and stiffness have been shown to promote malignant phenotypes and impact cellular response to chemotherapy treatment^52,53^. The importance of cell stiffness to biology, including resistance, has just been emerging in recent years^6–9^. Softer cancer cells are related to drug resistance by multiple mechanisms, including decreased sensitivity to apoptosis induction, enhanced metabolic activity, and regulation of key genes involved in extrusion of drugs^7^.

Connexins share structural features, consisting of one cytoplasmic N-terminus, two extracellular loops, one cytoplasmic loop, four transmembrane domains, and one C-terminal tail. Connexins mediated signaling via their C-terminal tails interacting with signaling mediators^22^. The C-terminal tail of CX43 has been found to interact with β-catenin, which reducing the amount of free β-catenin available to inhibit Wnt signaling and suppress cell proliferation^54^. On the other hand, CX43 also promote cancer migration *in vitro* by the C-terminal tail interacting with p38^55^. Actin and tubulin are known to be responsive to the C-terminal tail of CX43. It has been shown that CX43 can bind with microtubules and actin to regulate cytoskeleton rearrangements^23–27^, while the dynamic changes in the cytoskeleton are associated with cell stiffness^28^. Thus, these findings indicated that the aberrant expression of CX43 regulates cell stiffness in CRC. Indeed, our results showed that CX43 overexpressing could induce α-tubulin and F-actin polymerization and enhanced cell stiffness in CRC cells.

Stem cell-like cancer cells are a subset of cancer cells that can self-renew and are highly tumorigenic and are also regarded as the major responsible for drug resistance^56^. Recent studies have demonstrated that cellular softness may act as a biomechanical marker for cancer stem cells to better predict the prognosis of cancer patients^28,57^. Here, we provide further evidence that the stiff CRC cells mediated by CX43 overexpressing attenuate their stemness, leading to drug sensitivity, which suggests that physical properties of cancer cells might be targeted to overcome drug resistance. Together, these data suggest that the downregulation of CX43 in CRC increases the stemness of cells via reducing the cell stiffness, thus promoting the drug resistance.

## Conclusions

In summary, we determined that CX43 is capable of suppressing CRC progression. We also highlight that both effects, that is changes in the mechanical stiffness of the cell and GJIC mediated by CX43 deregulated, are closely related to drug resistance in CRC. Our results further suggest CX43 as potential biomarkers for cancer prognosis and as therapeutic targets against cancer growth and chemoresistance in CRC.

## Supplementary Material

Supplemental Material
