# Supplementary material for: CX43 down-regulation promotes cell aggressiveness and 5-fluorouracil-resistance by attenuating cell stiffness in colorectal carcinoma
Source: Cancer Biol Ther. 2023 Jun 21;24(1):2221879. doi: 10.1080/15384047.2023.2221879 (PMC10286671; doi:10.1080/15384047.2023.2221879)
Supplement: Supplemental Material [file KCBT_A_2221879_SM4308.docx]

**Supporting information**

**Table S1 primer sequences**

| name | sequences |
| --- | --- |
| CX43 | 5’-GAGTTTGCCTAAGGCGCTC-3’ |
|  | 5’-AGGAGTTCAATCACTTGGCG-3’ |
| Actin | 5’-TAAGGAGAAGCTGTGGTACG-3’ |
|  | 5’-GACTCGTCATACTCCTGCTT-3’ |

**Table S2 siRNA sequence of targeting Cx43**

| name | sequences |
| --- | --- |
| CX43-1 | 5’-GCUUAGAGUGGACUAUUAATT-3’ |
|  | 5’-UUAAUAGUCCACUCUAAGCTT-3’ |
| CX43-2 | 5’-GGGAAUCAAGCCAUGCUUATT-3’ |
|  | 5’-UAAGCAUGGCUUGAUUCCCTT-3’ |
| CX43-3 | 5’-GGUGGUAAUUGUGGCUAAATT-3’ |
|  | 5’-UUUAGCCACAAUUACCACCTT-3’ |
| CX43-4 | 5’-CGGUGGAGGUGGUACUCAATT-3’ |
|  | 5’-UUGAGUACCACCUCCACCGTT-3’ |
| Negative control | 5’-UUCUCCGAACGUGUCACGUTT-3’ |
|  | 5’-ACGUGACACGUUCGGAGAATT-3’ |

**Material and Methods**

**Cell proliferation and colony formation assays**

Cell proliferation was evaluated 5 day by the Cell Counting Kit (CCK-8) (Dojindo, Kumamoto,Japan) according to the manufacturer’s introductions. The colorectal cells were seeded and cultured in 96-well plates at 1 × 10^3^ cells per well. The plates were read on a microplate reader using a wavelength of 450 nm.

Colony formation assay were conducted in 6-well plates with cells seeded at 5 × 10^2^ cells per well and maintained in a complete medium for 2 weeks. Then, the cell colonies were fixed with methanol for 30 min and stained with Giemsa for 15 min. The surviving colonies (≥50 cells per colony) were then counted under a microscope. The experiments were performed at least in triplicate.

**Cell cycle and apoptosis analysis**

For cell cycle analysis, cells were harvested after 48 h transfection and fixed in ice-cold 70% ehanol at 4°C overnight. Cells were then resuspended in propidium iodide solution (Genechem, Shanghai, China) according to the manufacturer’s protocol and subjected to FACS analysis.

For apoptosis analysis, the cells were collected and washed with ice-cold PBS, resuspended in binding buffer and stained with Annexin V-FITC Detection Kit (Genechem) according to the manufacturer’s introduction. Analysis of cell apoptosis was carried out by using flow cytometry. The experiments were performed at least in triplicate.

**Wound-healing and transwell invasion assays**

Cell migration was assessed by measuring the movement of cells into a scraped area by a 10 μL pipette tube in 6-well plates. The spread of wound closure was observed every 24 h in serum-free medium, respectively. Migration was quantified by counting the distances of cells that migrated toward the original wound field.

Cell invasion assay was carried out by transwell chambers (BD Biosciences, San Jose, CA, USA) containing 8 μm pores in 24-well plates. 1 × 10^5^ cells were seeded into the upper matrigel-coated chambers in 200 μL of serum-free medium. The lower chamber was added with 500 μL of medium with 10% FBS. After incubated at 37 °C for 36 h, invading cells stuck to the lower surface of the membrane were fixed in 100% methanol for 30 min and stained with Giemsa solution for 15 min. The cells were counted under a microscope in 5 randomly selected fields. The experiments were performed at least in triplicate.

**Supporting figures**


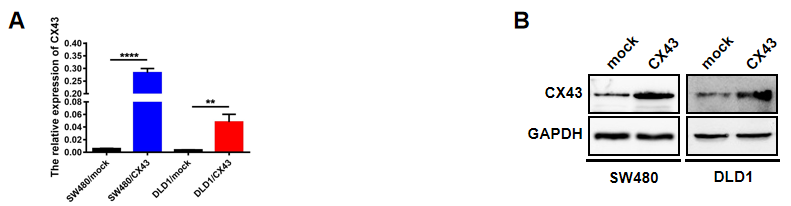


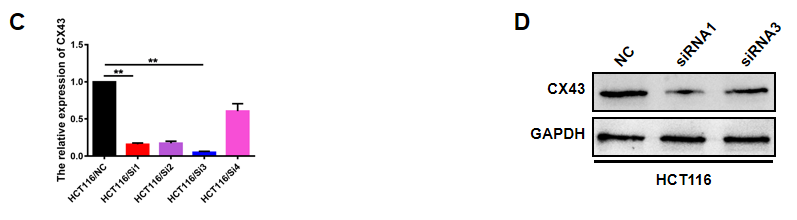


**Figure S1. The expression of CX43 after infection of lentivirus or transfection of siRNAs was confirmed in CRC cell lines by real-time RT-PCR and western blotting.**


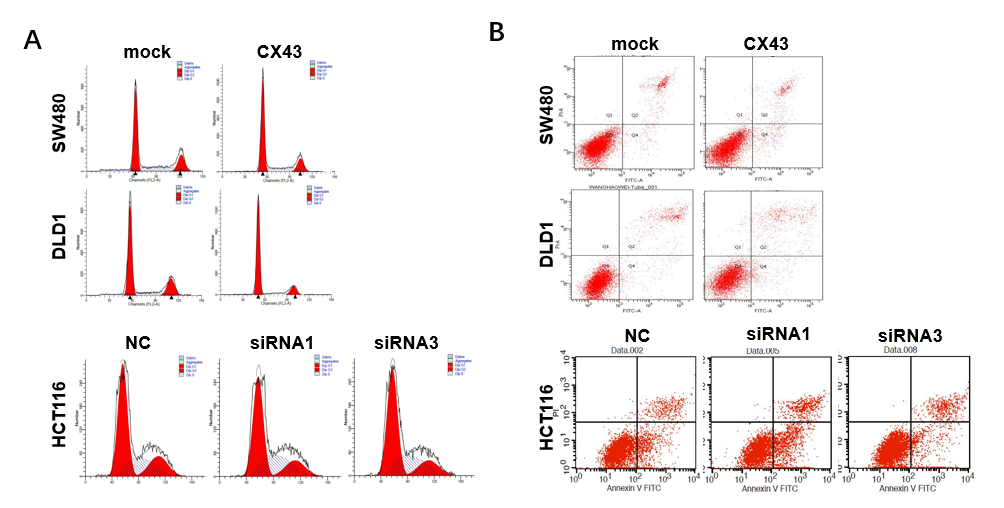


**Figure S2. The effect of CX43 on cell cycle and cell apoptosis in CRC cells.**


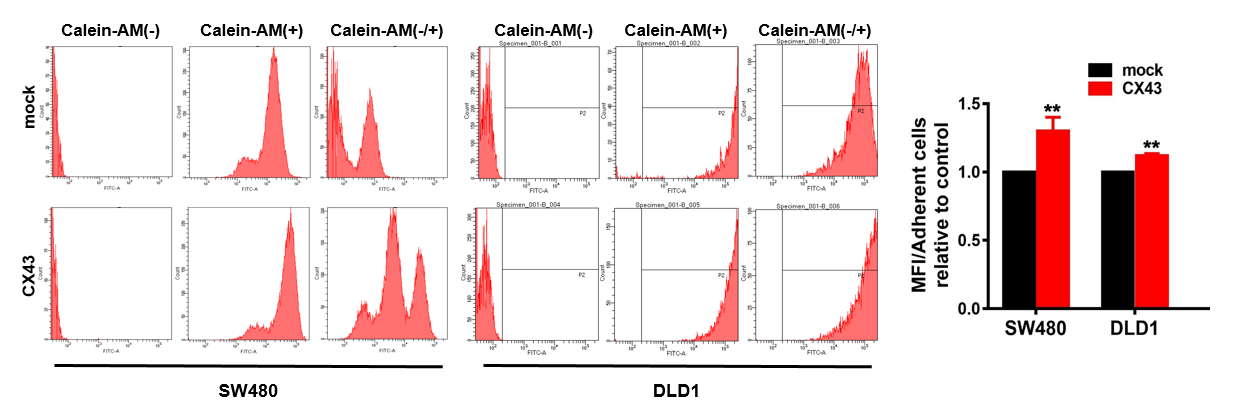


**Figure S3. Cx43 overexpressing increases cell-to-cell communication and cellular permeability.**


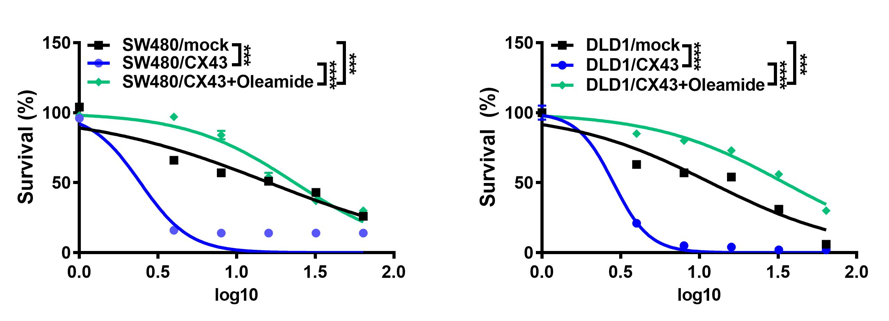


**Figure S4. Cx43 overexpressing increases cisplatin-sensitivity of CRC cells.**
